# Eculizumab in chemotherapy-induced thrombotic microangiopathy 

**DOI:** 10.5414/CNCS109836

**Published:** 2020-04-17

**Authors:** Lena Schulte-Kemna, Barbara Reister, Lucas Bettac, Ulla Ludwig, Daniel Fürst, Joannis Mytilineos, Carsten Bergmann, Rene van Erp, Bernd Schröppel

**Affiliations:** 1 Section of Nephrology, University Hospital, Ulm,; 2 Institute of Clinical Transfusion Medicine and Immunogenetics Ulm, German Red Cross Blood Transfusion Service, Baden Wuerttemberg-Hessen, and University Hospital Ulm,; 3 Institute of Transfusion Medicine, University of Ulm, and; 4 Medizinische Genetik Mainz, Limbach Genetics, Mainz, Germany

**Keywords:** thrombotic microangiopathy, complement, eculizumab, chemotherapy, remission, aHUS

## Abstract

Thrombotic microangiopathy (TMA) is a rare but severe complication of tumors and their chemotherapeutic treatment. We report on two patients with chemotherapy-induced TMA who were successfully treated with a short course of the terminal complement inhibitor eculizumab. Both patients quickly achieved remission of microangiopathic hemolytic anemia and recovery of renal function. After withdrawal of eculizumab, remission was stable over an observation period of 47 months and 15 months, respectively. Our data show that eculizumab is effective in treating chemotherapy-induced TMA. Discontinuation of eculizumab is feasible once the complement-activating condition is controlled and the trigger is eliminated. Additional studies need to determine the optimal duration of complement-directed therapies and validate effective monitoring strategies after discontinuation of such therapy.

## Introduction 

Thrombotic microangiopathy (TMA) encompasses a group of disorders presenting with microangiopathic hemolytic anemia (MAHA), thrombocytopenia, and ischemic organ damage, most frequently of the kidneys and the central nervous system [1, 2]. 

Atypical hemolytic uremic syndrome (aHUS) is a complement-mediated TMA caused by dysregulation of the alternative complement pathway. At least 50% of patients have an underlying inherited or acquired complement abnormality, exacerbated by complement-activating conditions, like infections, drugs, pregnancy, or cancer [3, 4]. 

Drug-induced TMA has been reported with antineoplastic agents including gemcitabine, docetaxel, and doxorubicin [5, 6, 7]. Direct cytotoxic and immune-mediated endothelial damage have been proposed as underlying pathologies [5]. While immune-mediated damage generally shows acute onset within 2 – 3 weeks of drug exposure, the clinical manifestation of cytotoxic damage is either acute or slowly progressive with cumulative dose-dependent toxicity [5, 8, 9]. Distinguishing cancer-related TMA as a consequence of cancer itself from cases of chemotherapy-induced TMA can be challenging. However, metastatic disease is more common in cancer-related TMA, whereas in chemotherapy-induced TMA, little or no active malignancy is detectable [10]. While discontinuation of the offending drug and supportive care are the primary treatment options in drug-induced TMA, in some cases this intervention is unable to limit the already dysregulated complement activity and requires therapeutic complement inhibition. 

Eculizumab, approved as therapy for aHUS in 2011, is a humanized monoclonal antibody that binds to the complement component C5, preventing its cleavage into C5a and ultimately the formation of the membrane attack complex (SC5b-9) [11]. 

While the contributory role of complement dysregulation in drug-induced TMA is increasingly acknowledged, data on the efficacy of eculizumab, the duration of such therapy, and the incidence and type of detected complement abnormalities are sparse. 

Here, we report on two patients with chemotherapy-induced TMA, who were successfully managed with temporary eculizumab therapy and remained relapse free for a follow-up of 47 and 15 months, respectively. [Table Table1]

## Case reports 

### Patient 1 

A 52-year-old woman admitted with acute onset of altered mental status, bloody diarrhea, and anuric acute kidney injury. Six months prior to admission, chemotherapy with docetaxel, doxorubicin, and cyclophosphamide was started for invasive ductal breast cancer. The last dose of chemotherapy was administered 3 days before symptoms started. Admission laboratory showed serum creatinine of 480 µmol/L, Coombs-negative hemolytic anemia with schistocytes on peripheral blood smear and thrombocytopenia of 64/µL. Lactate dehydrogenase (LDH) was 1,658 IU/mL and haptoglobin < 0.10 g/L. Coagulation tests were within the normal range, international normalized ratio (INR) 1.3 and partial thromboplastin time (PTT) 35 seconds, ruling out disseminated intravascular coagulation. Shiga toxin producing *E. coli *associated hemolytic uremic syndrome (STEC-HUS) was excluded by negative stool cultures for Shiga toxin-producing *E. coli* strains. Stool cultures for shigella, salmonella, campylobacter, and yersiania as well as PCR for *Clostridium difficile* were negative. Thrombotic thrombocytopenic purpura (TTP) was excluded by ADAMTS13 activity of 37% of control values. No ADAMTS13 antibodies were detected. 

**Figure 1.**
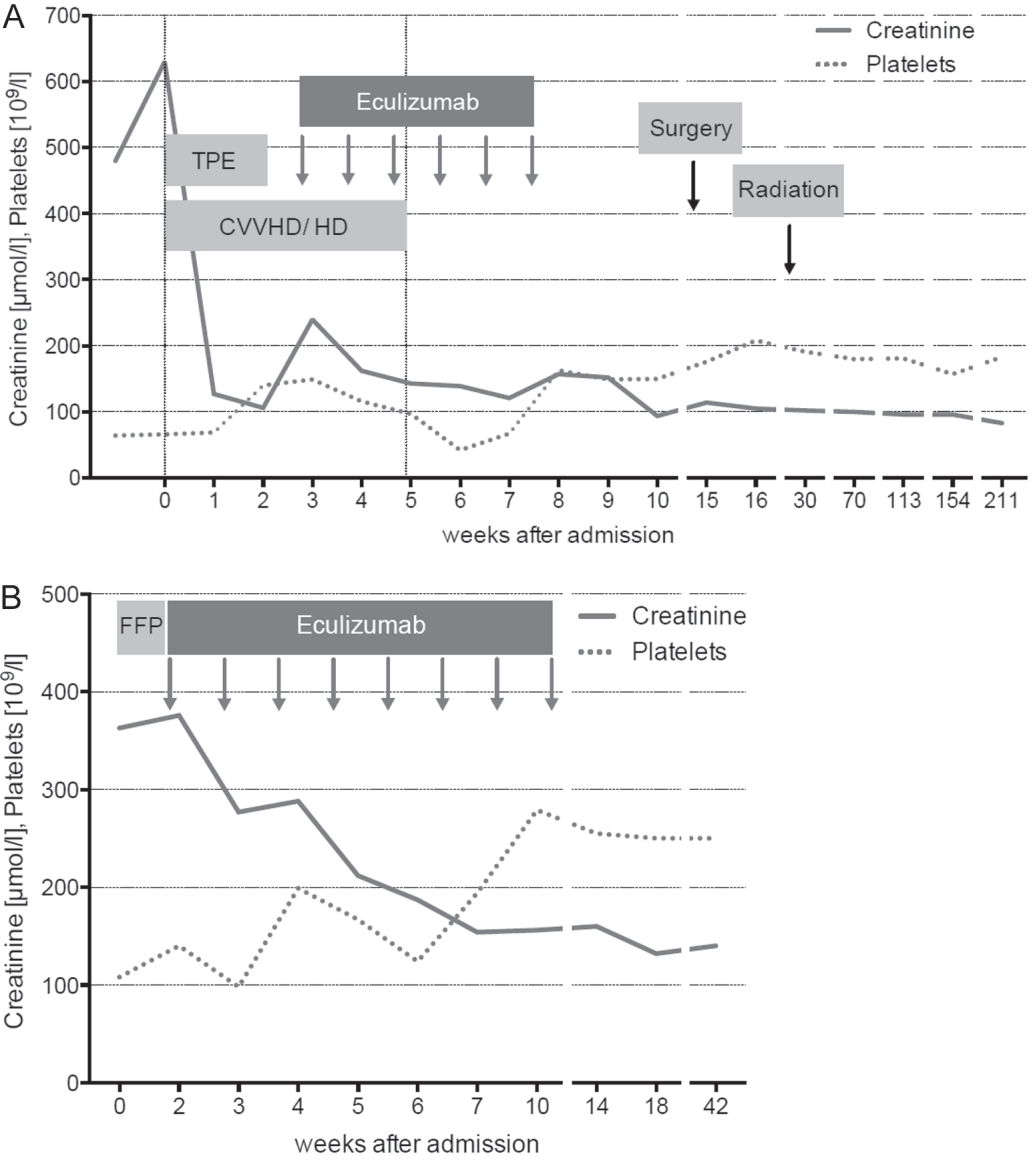
A: Case 1, induction therapy with 6 doses of eculizumab. Serum creatinine and thrombocytes from admission to last follow-up (week 211). Breast-conserving surgery was performed 7 weeks after withdrawal of eculizumab, followed by radiation therapy 3 months later. TPE = therapeutic plasma exchange; CVVHD = continuous veno-venous hemodialysis; HD = hemodialysis. B: Case 2, induction therapy with 8 doses of eculizumab. Serum creatinine and thrombocytes from admission to last-follow up (week 42). FFP = fresh frozen plasma.

Shortly after admission, she developed seizures with respiratory failure requiring intubation. With the diagnosis of TMA, therapeutic plasma exchanges (TPE) were started. Daily TPE over 12 days and steroid therapy showed no effect on clinical symptoms and hemolysis. She had persistent seizures and required renal replacement therapy. Eculizumab was eventually initiated 20 days after admission. Immediately after the first dose of eculizumab, we observed a rapid and dramatic improvement of neurological symptoms. Renal replacement therapy could be discontinued 2 weeks later. Eculizumab was administered 6 times over a period of 5 weeks (Figure 1A). Breast-conserving surgery was performed 7 weeks after termination of eculizumab, followed by radiation therapy. During 47 months of follow-up, renal function continued to improve (eGFR 68 mL/min), and no relapse of TMA has occurred (Figure 1A) (Table 2). 

Next-generation sequencing identified a homozygous polymorphism in complement factor H (CFH) gene (synonymous variant c.1419 G>A, p.Ala473Ala). CFH autoantibodies were not detected. 

### Patient 2 

A 57-year-old woman admitted with hypertensive urgency, progressive decline of renal function, and MAHA. She was diagnosed with pancreatic cancer 30 months prior to admission and since then treated with gemcitabine and Nab-paclitaxel. Chemotherapy had been discontinued 6 weeks earlier when a decline in renal function, hemolytic anemia, and mild thrombocytopenia (130/µL) was first noted. 

Admission laboratory showed serum creatinine of 363 µmol/L (eGFR 11 mL/min), Coombs negative hemolytic anemia with schistocytes on peripheral blood smear, and thrombocytopenia of 108/µL. LDH was 760 IU/mL, haptoglobin < 0.10 g/L, and coagulation tests were within the normal range (INR 1, PTT 31 seconds). Stool cultures were negative for Shiga toxin-producing *E. coli*, and ADAMTS13 activity was 61% of control values. Urinalysis showed proteinuria with a protein/creatinine ratio of 0.8 g/g. Several plasma infusions were given without improvement of hemolysis or renal function. The patient received eculizumab 7 days after admission, followed by prompt resolution of hemolysis and improvement of renal function. Eculizumab was discontinued after a total of 8 doses over a period of 10 weeks. During 15 months of follow-up, renal function remained stable (eGFR 36 mL/min), and no relapse of TMA occurred (Figure 1B) (Table 2). The patient died of pancreatic cancer 18 months after initial hospital admission. 

Next-generation sequencing identified a heterozygous polymorphism in CFH gene (synonymous variant c.1419 G>A, p.Ala473 Ala). 

## Discussion 

We report on two patients with chemotherapy-induced TMA, persistent after discontinuation of the culprit drug and TPE/plasma infusion. In both patients, the clinical response to therapy with eculizumab was prompt and remission stable after cessation of treatment. Treatment with eculizumab was well tolerated, and no adverse events were reported. 

Due to limited clinical experience, the optimal strategy for treatment of chemotherapy-induced TMA, especially the role of eculizumab, is not yet clear. Discontinuation of the offending drug and supportive care are the primary treatment options. Due to the long turnaround time for us to receive the results of the ADAMTS13 activity plasma infusions (case #2) and therapeutic plasma exchange (case #1) was initially used. 

In agreement with others we found that TPE/plasma infusion was not effective in patients with chemotherapy-induced TMA [12, 13, 14, 15, 16, 17, 18]. 

Patient 1 presented, among other symptoms, with bloody diarrhea. However, the presence of diarrhea is not sufficient to exclude other forms of TMA as ~ 30 – 40% of aHUS and TTP cases involve gastrointestinal symptoms, including bloody diarrhea [19, 20]. 

Genetic mutations leading to dysregulation of the alternative complement pathway or autoantibodies against complement regulatory proteins are identified in ~ 50% of aHUS patients [4]. However, genetic variants in complement regulatory proteins are also detected in patients with secondary, e.g., chemotherapy-induced, TMA [3]. In such patients, the underlying dysregulation of the alternative complement system may be unmasked by the applied drug, acting as a complement-activating trigger. Given the incomplete penetrance of the genetic defects, complement-activating conditions play an important role for the development of TMA [3, 21, 22]. 

In most patients with TMA, a complement-activating trigger can be identified, and in 28% of patients with an activating trigger, a genetic risk mutation can be found [3]. 

The largest group of aHUS-associated mutations occurs in the CFH gene, and more than 60% of these mutations are clustered within the C-terminal recognition region [23, 24, 25]. CFH is a central regulator of the alternative pathway of complement by acting as a cofactor to factor I in the breakdown and inactivation of C3b [26]. 

In both of our patients, we identified a polymorphism in the CFH gene (c.1419 G>A). This variant has a high allele frequency in the general population, and its isolated occurrence seems not to be associated with an increased aHUS risk [27]. 

The mechanism of gemcitabine-related TMA appears to be dose-related with a reported incidence of 1%. Both immune-mediated and cytotoxic injury have been proposed as underlying pathophysiology [12, 28]. Cytotoxic damage is the assumed mechanism in the few described cases of doxorubicin- and docetaxel-related TMA [5, 6, 29]. 

To date, several cases of the use of eculizumab in chemotherapy-induced TMA have been reported, most of them regarding gemcitabine (summarized in Table 1). Before the availability of eculizumab, a case series reported ~ 29 patients with suspected gemcitabine-related TMA. Despite discontinuation of gemcitabine, 7 (24%) patients progressed to end-stage renal disease (ESRD), and 3 (10%) patients developed chronic renal failure [30]. These reports and our observation support induction therapy with eculizumab in cases of persisting TMA. 

Eculizumab is approved for lifelong therapy of aHUS. However, the possible side effects, especially the risk of meningococcal infection, the inconvenience of a bi-monthly application, and the significant costs have prompted interest in alternative dosing schedules and complete discontinuation. A recent review analyzed data from unpublished cases, published case reports, clinical trials, and the Global aHUS Registry regarding patient outcomes after eculizumab discontinuation [31]. Of the case reports, a subsequent TMA manifestation was observed in 31% (16/52) of patients after eculizumab discontinuation. Data from five clinical trials documented a relapse in 20% (12/61) of patients after cessation of therapy with eculizumab with a median follow-up of 24 weeks. Terminal renal failure occurred in 5% (3/61) of the patients. Of note, relapse risk was independent of an identified genetic mutation, high-risk polymorphism, or autoantibody status. Data from the Global aHUS Registry found a relapse in 16% (12/76) of patients. In the cases described above, disease recurrence was unpredictable in both timing and severity [31]. 

The French aHUS Registry described a relapse rate after eculizumab discontinuation in 31% (12/38) of the patients [32]. The risk of recurrence was higher in the presence of complement gene variants. The highest risk was associated with CFH variants, whereas no relapse was seen in patients without identified mutations or negative CFH autoantibodies. In case of relapse, early reinstitution (≤ 48 hours) of eculizumab resulted in rapid hematologic remission and a return of serum creatinine to baseline level [32]. 

While current evidence suggests a relapse rate after eculizumab discontinuation of ~ 30%, there is little available clinical data for estimating the risk of relapse in chemotherapy-induced TMA [29]. 

In 2017, the KDIGO controversies conference published recommendations for best treatment strategies in aHUS. No evidence was currently seen to support lifelong therapy in all aHUS patients. The consensus suggested that eculizumab withdrawal could be considered on an individual and risk-stratified basis after a minimum treatment duration of 6 – 12 months to ensure recovery of endothelial damage [33]. Important risk factors for TMA relapses constitute an identified genetic mutation, former TMA episodes, or concomitant permanent or likely recurrent complement-activating condition. Close monitoring of renal function and hematological parameters after eculizumab withdrawal is mandatory; however, there are no evidence-based data about the reliability of a specific parameter and the optimal frequency of testing [33]. 

In summary, our report supports the role of complement-directed therapy with eculizumab as an effective therapeutic option in the management of refractory chemotherapy-induced TMA. In our opinion, eculizumab discontinuation is feasible in carefully selected patients after permanent removal of the complement-activating condition. Further studies are needed to elucidate the role of genetic variants in complement-regulatory proteins in chemotherapy-induced TMA and to define parameters predictive of complement activation and likely TMA recurrence. Until then, the decision to withdraw eculizumab has to be made on an individual basis. 

## Funding 

None. 

## Conflict of interest 

B.S.: Consultant/Speaker Honoraria from Alexion, Amgen, Novartis, Astellas, Boehringer Ingelheim, Vifor Pharma, Astra-Zeneka, Janssen. Grants from Alexion, Sanofi, Pfizer. 

C.B. is an employee of Limbach and holds a part-time faculty appointment at the University of Freiburg. His research lab receives support from the Deutsche Forschungsgemeinschaft (DFG) DFG BE 3910/8-1 and DFG BE 3910/9-1, the Collaborative Research Center (SFB) KIDGEM 1140 and from the Federal Ministry of Education and Research (BMBF, 01GM1903I and 01GM1903G). He received speaker honoraria from Alexion and PTC Therapeutics. 

The other authors do not have a conflict of interest. 

**Table 1. Table1:** Studies of chemotherapy-induced thrombotic microangiopathy treated with eculizumab.

Patients (n)	Drug	Previous therapy	Doses of eculizumab (range) / duration of treatment	Median follow-up (range)	Improved renal outcome	Genetic analysis	Reference
1	Gemcitabine	DW + TPE + Steroids + RTX	4 / 3 weeks	17.5 weeks	Yes	NT	Starck [34] 2014
1	Mitomycin C	DW + TPE	8 / 3 months	18 months	Yes	NT	Faguer [35] 2013
1	Cisplatin	DW	Not reported / 4 months	Not reported	Yes, relapse 2 months after stop of eculizumab	CD46 mutation	Gilbert [36] 2013
4	Gemcitabine	DW + TPE in 1 patient DW + steroids in 1 patient DW in 2 patients	6.25 (5 – 8) / not reported	Not reported	Yes	NT	Al-Ustwani [37] 2014
1	Gemcitabine	DW + TPE	4 / not reported	11 weeks	No	ND	Tsai [38] 2014
1	Gemcitabine	DW + Steroids	6 / 7 weeks	3 months	No	NT	Karkowsky [39] 2015
1	Gemcitabine	DW + TPE	7 / 10 weeks	Not reported	Yes	NT	Rogier [16] 2016
1	Gemcitabine	DW + TPE	7 / 8 weeks	3 months	Yes	NT	Lopez [40] 2017
8	Gemcitabine	DW	4.5 (3 – 22) / not reported	Not reported	Yes	NT	Grall [41] 2016
7	Gemcitabine Dasatinib Bevacizumab Bleomycin	DW DW + TPE (2 patients)	Not reported / 14 weeks (2 – 24 weeks), 1 ongoing	Not reported	Yes	NT	Weitz/Deloughery [42] 2018
2	Gemcitabine Carfilzomib	DW + TPE	Not reported	42.5 weeks (33-52)	Yes	NT	Gosain [43] 2017
1	Gemcitabine	DW + TPE	20 / 9 months	17 months	Yes	NT	Krishnappa [44] 2018

DW = offending drug withdrawn; TPE = therapeutic plasma exchange; RTX = rituximab; NT = not tested; ND = not detected.

**Table 2.** Summary case report 1 and 2.

Case	Age/ Gender	CAC	Organ involvement/ Presentation	Genetic	Eculizumab duration/doses	Creatinine (µmol/L) at the end of eculizumab	Creatinine (µmol/L) at last follow-up	Relapse	Follow-up (months)
1	52 y/F	Docetaxel Doxorubicin	Kidney/acute CNS/acute Lungs/acute	CFH polymorphism synonymous variant c.1419 G>A, p.Ala4773 Ala	5 weeks/6	120	83	No	47
2	57 y/F	Gemcitabine	Kidney/chronic Severe hypertension	CFH polymorphism Synonymous variant c.1419 G>A p.Ala473 Ala	10 weeks/8	154	140	No	15

CAC = complement activating condition; CNS = central nervous system; CFH = complement factor H.
